# Dietary Patterns and Food Choices of Adults With Inflammatory Bowel Disease in New Zealand

**DOI:** 10.1002/jgh3.70457

**Published:** 2026-08-09

**Authors:** Peter Vivian Acire, Angharad Vernon‐Roberts, Andrew S. Day

**Affiliations:** ^1^ Department of Paediatrics and Child Health University of Otago Christchurch Christchurch New Zealand

**Keywords:** dietary patterns, disease activity, food choices, health‐related quality of life, inflammatory bowel disease

## Abstract

**Aims:**

This study aimed to assess current dietary patterns in adults with inflammatory bowel disease (IBD) in New Zealand and to evaluate associations with current self‐reported disease activity and well‐being.

**Methods:**

A prospective online survey was conducted using validated dietary data collection instruments. Dietary patterns were derived using principal component analysis. Self‐reported current disease activity and health‐related quality of life (HRQoL) scores were ascertained. Relationships between diet patterns and other variables were assessed.

**Results:**

The responses of 205 participants were included in the analysis: mean age 43.0 (±14.0) years, and a mean disease duration of 31.5 (±14.0) years. There were 107 (52%) people with Crohn's disease (CD) and 98 (48%) with ulcerative colitis/IBD‐unclassified (UC/IBDU). Six dietary patterns were identified among the cohort data: Western, vegetarian, pescatarian, semi‐vegetarian, semi‐pescatarian, and low‐carbohydrate. Consuming a Western dietary pattern was associated with active CD (adjusted odds ratio [AOR] = 4.55, 95% CI [1.27, 7.26], *p* = 0.02) or active UC/IBDU (AOR = 3.50, 95% CI [1.07, 5.40], *p* = 0.04). In contrast, people with CD following a vegetarian diet pattern were less likely to report active disease (AOR = 0.32, 95% CI [0.11, 0.98], *p* = 0.04). Adherence to a Western or semi‐pescatarian dietary pattern was predictive of worse HRQoL.

**Conclusions:**

In this group of adults with IBD, vegetarian dietary patterns were associated with lower current CD activity but not UC/IBDU, while the Western dietary pattern was associated with higher disease activity and impaired HRQoL.

## 1 Introduction

Inflammatory bowel disease (IBD) is a progressive, debilitating, and chronic inflammatory disorder of the gastrointestinal (GI) tract that includes Crohn's disease (CD) and ulcerative colitis (UC) [1]. The rates of IBD have increased globally over the last few decades [2], with an estimated 4.9 million individuals with IBD worldwide in 2019 [3]. Furthermore, rising incidence has been reported in many countries, such as in Latin America and Asia [4, 5]. A recent systematic review reported an increased IBD prevalence rate of 303.3 per 100 000 persons in the Oceania region among adults [6], and 36.0 per 100 000 persons among children [7]. In the Canterbury region of New Zealand (NZ), one in 150 persons have IBD, making it one of the regions in the world with the highest IBD prevalence [8].

IBD can cause significant morbidity that may lead to a substantial burden on healthcare systems [9, 10, 11, 12]. Moreover, increased disease activity is associated with impaired health‐related quality of life (HRQoL) [13]. In addition, inadequate or incomplete management of active disease may lead to chronic and irreversible bowel damage, such as strictures in CD, requiring surgical intervention [14]. In children, IBD can also impair growth, development, and quality of life [15].

While the etiology of IBD is multifactorial, dietary aspects appear to play a key role [16]. Diet not only contributes to the development of IBD but is also crucial for disease management after diagnosis [17]. Observational studies and randomized controlled trials (RCTs) have shown strong correlations between dietary patterns and IBD risk, disease activity, and HRQoL [18, 19, 20]. The Western dietary pattern, characterized by intake of high‐fat and high‐sugar foods, has been shown to almost double the risk of developing IBD, thereby likely contributing to the increasing rates in recent years [21]. Conversely, plant‐based food consumption patterns may contribute to the induction or maintenance of remission [22]. Additionally, dietary modification can prevent or improve symptoms in more than two‐thirds of patients with IBD [23, 24, 25].

Studying dietary patterns in the IBD setting is important because nutrients often act synergistically or antagonistically as part of a whole meal or daily dietary intake [26]. In addition, because habitual dietary intake and its relative impact may differ between individuals and contexts [27], there is a need to understand this in the local setting. There is currently limited data regarding dietary patterns and food choices among people with IBD, including in NZ. Thus, the primary goal of this study was to assess the current dietary patterns and food choices of a group of adults with IBD in NZ. Secondary aims were to consider associations between dietary patterns and self‐reported disease activity and HRQoL.

## 2 Methods

### 2.1 Study Design

This was a prospective cross‐sectional survey involving adults with IBD from NZ. The study was carried out between July 1, 2025, and September 30, 2025.

### 2.2 Participants and Recruitment

People were eligible to participate if they were adults diagnosed with IBD (CD, UC or IBD‐unclassified [IBDU]). Participants were excluded if they reported that they had been diagnosed with IBD but did not permanently reside in NZ.

Participants were invited to take part in the survey via the Facebook and X (formerly Twitter) social media platforms of Crohn's and Colitis NZ (CCNZ). Invitations to participate were circulated twice via newsletter over 6 weeks and also published on the CCNZ website.

### 2.3 Survey Tool

The entire survey document consisted of 139 questions, answered as checkboxes, radio buttons, and sliding Visual Analogue Scales (VAS). While the majority of the questions were in multiple‐choice format, some (on food choices) required free‐text responses.

Initial general questions focused on personal background and disease factors. Individual sections then centered on specific variables using validated assessment methods: these included body mass index, dietary patterns, current disease activity, and HRQoL.

The survey was piloted with members of the Department of Pediatrics and Child Health at the University of Otago, Christchurch, to assess readability and understanding. All data were collected via the Qualtrics XM survey tool (Qualtrics, Provo, USA).

### 2.4 Background Information

Preliminary questions were directed to collect key demographic data: sex, age, marital status, place of residence, ethnicity, education history, and current employment. For place of residence, participants were grouped according to the four Health New Zealand regional groupings (i.e., Northern, Midland, Central, and the South Island) [28]. Participants were then asked to provide details of their IBD, including disease classification, disease duration, existing comorbidities, and current medical therapies.

### 2.5 Self‐Reported Anthropometry

Participants were asked to self‐report their current weight (kilograms) and height (centimeters) for computing their body mass index (BMI) (kg/m^2^). The World Health Organization (WHO) BMI cutoffs for adults were used: < 18.5 was categorized as underweight, BMI 18.5–24.9 as normal, BMI ≥ 25 as overweight, and BMI ≥ 30 as obese [29].

### 2.6 Dietary Patterns and Food Choices

Participants were asked to report details of their eating frequency and number of servings for each food item they had eaten in the past month using the Researching, Eating, Activity, and Cognitive Health Food Frequency Questionnaire (REACH‐FFQ) [30]. The REACH‐FFQ is a 109–item semi‐quantitative tool developed and validated in NZ to assess dietary intake in adults. It uses serving sizes based on guidance from FOODfiles, a NZ food composition database [31]. For each food item, it asks the frequency of consumption and serving size over the past months, with 10 response choices. To minimize recall bias, participants were asked about their consumption frequency over 3 months.

The REACH‐FFQ was supplemented with eight open‐ and closed‐ended questions to capture additional information on oral vitamin/mineral supplement intake and current dietary intervention(s). Participants were also asked to list any foods or drinks that they have noted that improve or aggravate their IBD symptoms.

### 2.7 Self‐Reported Disease Activity

The Simplified Crohn's Disease Activity Index (SCDAI) was utilized to provide self‐reported current disease activity in the respondents with CD [32]. Scores < 5 points were indicative of remission, while those between 5 and 7 indicated mildly active disease, 8–16 points moderately active disease, and > 16 points as severe disease.

The current disease activity for subjects with UC or IBDU was ascertained using the Simplified Clinical Colitis Activity Index (SCCAI) [33]. Scores ranged from 0 to 19, where an SCCAI score < 5 represented inactive disease and ≥ 5 represented active disease.

### 2.8 Health‐Related Quality of Life

The Short Health Scale (SHS) was employed to provide HRQoL data [34]. The SHS is an IBD‐specific tool comprised of a four‐item questionnaire that has been validated and widely used in children and adults with IBD. Participants rated the disease impact based on the self‐assessment of four aspects of their health: symptoms, function, disease‐related worry, and general well‐being. Responses were graded on a 100‐mm VAS correlating with a score range of 0–100, with higher scores indicating worse HRQoL. Scores for each of the four dimensions and the total SHS score were obtained.

### 2.9 Data Analysis

Data were analyzed using SPSS Statistics for Mac OS, Version 30.0 (IBM, Armonk, NY). Respondent characteristics were presented using descriptive statistics. Normally distributed continuous variable data were presented as means with corresponding standard deviations (SD), while the median with interquartile range (between the 25th and 75th percentile [IQR]) was used for non‐normally distributed data. All categorical variables were presented as numbers with corresponding percentages. Comparisons between categorical variables were computed using the *χ* ^2^ test. Nonparametric tests (Mann–Whitney *U* test for two independent groups and Kruskal–Wallis *H* test for two or more groups) were performed. For continuous variables with normally distributed data, comparisons were made using the independent sample *t*‐test.

For the free‐text responses on food choices, manifest content analysis was implemented using R text mining and Wordcloud package, version 4.5.2 (R Foundation for Statistical Computing, Vienna, Austria). Word frequencies and percentages for the listed foods and drinks were generated. The first 10 foods and drinks (i.e., items with higher frequency counts) were selected and presented as bar plots.

Principal component analysis (PCA) was conducted to identify dietary patterns [35]. PCA is a data‐driven method that derives dietary intake patterns from population data through dimensionality reduction techniques. PCA is advantageous over other dietary pattern analysis methods because it enables the description of variation in dietary intake and evaluation of the overall dietary quality of a population, with good reproducibility [35].

Prior to analysis, the 109 food items were categorized into 36 food groups. The food items were grouped according to their nutrient profile and type, culinary value, and NZ food data (Table S1) [36]. Food grouping was also based on a previous study that employed the same tools [37]. Foods consumed by less than 10% of individuals were excluded from the analysis (Table S2) [38]. Before PCA was performed, food frequency entries were converted into a single and consistent measure (daily consumption). *Z*‐score normalization was conducted to standardize the data to minimize skewness.

The PCA orthogonal (varimax) rotation was executed using SPSS. The Kaiser–Meyer–Olkin (KMO ≥ 0.60) and Barlett's test of sphericity were used to ascertain sampling adequacy and correlations between items for further PCA [39]. An eigenvalue of value > 1 and a break in the scree plot were used to ascertain the number of dietary patterns to retain [40]. Food variables with a factor loading ≥ 0.30 were included in the component matrix (i.e., the more positive the factor loading, the closer the food variables to the dietary pattern) [41]. Each participant's dietary pattern score was calculated by totalling the standardized daily consumption weighted by the respective regressed factor loadings [42].

Binary and multinomial logistic regression analyses were performed to determine associations between the PCA‐identified dietary patterns and the relevant disease activity groupings. Analysis of the relationship between dietary patterns and HRQoL was conducted using multivariate multiple linear regression. Potential confounding variables were added in the regression models as covariates in a stepwise manner. Variables that changed the coefficient (*B*) of the main exposure by more than 10% were considered confounders. All assumptions were tested and met before the analysis. Variables were checked for the presence of collinearity, with a variance inflation factor (VIF) between 1 and 10 used to show the absence of collinearity. Significant Omnibus tests of model coefficients (*p* < 0.05) and non‐significant Hosmer–Lemeshow goodness‐of‐fit test (*p* > 0.05) were used to assess the model fit. A two‐sided *p*‐value of < 0.05 was considered significant.

## 3 Results

### 3.1 Study Participants

Two hundred and twenty‐four participants responded to the survey. As the number of people who received or viewed the invitation to participate was unable to be defined, the exact study response rate was not definable.

Nineteen (8%) of the 224 respondents were excluded due to incomplete data, leaving 205 participants (107 (52%) CD; 98 (48%) UC/IBDU) in the final analysis (Table 1). There were more female participants (*n* = 179; 87%) than males. The mean age of the participants with CD was 41.5 (±13.2) years and 44.9 (±14.7) years for those with UC/IBDU. Approximately one quarter of the participants (*n* = 54; 26%) lived in the Canterbury region of the South Island of NZ.

**TABLE 1:** Demographic and disease characteristics of 205 adults with IBD who provided data in response to the study invitation.

Variable				
		CD (*n* = 107) *N* (%)	UC/IBDU (*n* = 98) *N* (%)	All IBD (*n* = 205) *N* (%)
Sex	Male	13 (12)	12 (12)	25 (12)
	Female	94 (88)	85 (87)	179 (87)
	Nonbinary	0 (0)	1 (1)	1 (1)
Age, mean (SD)		41.5 (±13)	44.9 (±15)	43.2 (±14)
Marital status	Single	24 (22)	16 (16)	40 (20)
	Married	49 (46)	48 (49)	97 (47)
	Divorced/widowed/separated	6 (6)	8 (8)	14 (7)
	Informally married/cohabiting	24 (22)	24 (26)	48 (23)
	Prefer not to say	4 (4)	2 (2)	6 (3)
Residence	Northern	11 (10)	17 (17)	28 (14)
	Midland	23 (22)	9 (9)	32 (16)
	Central	25 (2)	25 (26)	50 (24)
	Canterbury	31 (29)	23 (24)	54 (26)
	The rest of the South Island	17 (16)	24 (24)	41 (20)
Ethnicity	NZ European	98 (91)	90 (92)	188 (92)
	Māori	4 (4)	8 (8)	12 (6)
	Asian	5 (5)	0 (0)	5 (2)
Highest level of education attained	High School Level	13 (12)	15 (15)	28 (14)
	National certificate/diploma	38 (36)	23 (23)	61 (30)
	Bachelor's degree or graduate certificate/diploma	32 (30)	29 (30)	61 (30)
	Postgraduate study (PGCert/Dip, Master's)	19 (20)	26 (26)	45 (22)
	Prefer not to say	4 (4)	5 (5)	9 (4)
Employment	Paid employment	66 (62)	63 (64)	129 (63)
	Self‐employment	12 (11)	10 (10)	22 (11)
	Currently not working	9 (8)	13 (13)	22 (11)
	Volunteer	3 (3)	2 (2)	5 (2)
	Other (pensioner/retired or student)	17 (16)	10 (10)	27 (13)
Disease duration, mean (SD±)	Disease duration (years)	29.6 (±13.6)	33.4 (±14.3)	31.5 (±14.0)
SCDAI	Remission (< 5)	47 (44)	—	—
	Mild disease (5–7)	30 (28)	—	—
	Moderate disease (8–16)	30 (28)	—	—
	Severe (> 16)	0 (0)	—	—
SCCAI	Inactive disease (< 5)	—	52 (53)	—
	Active disease (≥ 5)	—	46 (47)	—
Existing comorbidity^a^	Obesity	18 (17)	8 (8)	26 (13)
	Coeliac disease	18 (17)	9 (9)	27 (13)
	Arthritis	16 (15)	15 (15)	31 (15)
	None	29 (27)	44 (46)	73 (36)
	Other	26 (24)	22 (22)	48 (23)
Medication type^a^	Aminosalicylates	10 (9)	24 (26)	34 (17)
	Corticosteroids	13 (12)	8 (8)	21 (10)
	Immunosuppressants	19 (18)	9 (9)	28 (14)
	Biologics	39 (36)	21 (21)	60 (29)
	JAK inhibitors	0 (0)	21 (21)	21 (10)
	No current medication	11 (10)	13 (13)	24 (12)
	Other	15 (14)	2 (2)	17 (8)

*Note:* Other comorbidities included Gilberts Syndrome, Polycystic Ovarian Syndrome, Migraines, Vertigo, Serrated Polyposis Syndrome (SPS), Endometriosis, Fibromyalgia, Asthma, Irritable Bowel Syndrome, Osteoporosis, Hypertension, Kidney Stones, Graves' disease, Central Pain Syndrome, Postural Orthostatic Tachycardia Syndrome, Premenstrual dysphoric disorder (PMDD), Sarcoidosis, and Mental health disorders. The variables are number (%) or mean (SD±).

Abbreviations: CD, Crohn's disease; IBDU, inflammatory bowel disease unclassified; JAK, Janus kinase; *n*, number; PGCert/Dip, postgraduate certificate/diploma; SCCAI, Short Clinical Colitis Activity Index; SCDAI, Short Crohn's Disease Activity Index; SD, standard deviation; UC, ulcerative colitis.

^a^ Participants could report more than one.

The mean disease duration was 29.6 (±13.6) years for those with CD and 33.4 (±14.3) years for participants with UC/IBDU. Forty‐seven (44%) participants with CD were in (self‐reported) remission, and more than half (*n* = 52; 53%) of those with UC/IBDU had inactive disease.

Seventy‐eight (73%) individuals with CD reported having one or more comorbidities, while more than half (*n* = 54; 55%) of those with UC or IBDU reported this. The most commonly reported comorbidities overall were obesity (*n* = 22; 21%), coeliac disease (*n* = 21; 20%), and arthritis (*n* = 24; 23%). Of the 205 respondents, 181 (88%) reported current medical therapies. Twenty‐four (12%) participants did not report any current use of medication.

### 3.2 Participants' Nutritional Status, Oral Supplement Use, and Specific Dietary Intake

The median BMI of the cohort with CD was 26.8 kg/m^2^ (IQR 23.4–30.7) and 24.3 kg/m^2^ (IQR 21.2–29.4) for those with UC/IBDU (*p* = 0.025) (Table 2). The combined proportion of overweight and obese respondents (*n* = 110; 53%) was similar to that for the normal and other lower BMI categories (*n* = 95; 47%).

**TABLE 2:** Current medication use, nutritional status, oral supplements, and specific dietary intake of 205 adults with IBD.

		CD or UC/IBDU		All IBD (*n* = 205) *N* (%)
		CD (*n* = 107) *N* (%)	UC/IBDU (*n* = 98) *N* (%)	
BMI (kg/m^2^), median (IQR)		26.8 (23.4–30.7)	24.3 (21.2–29.4)	25.7 (22.8–30.1)
Nutritional risk categorized by BMI	Underweight (≤ 18.5)	4 (4)	5 (5)	9 (5)
	Normal (18.5–24.9)	41 (38)	45 (46)	86 (42)
	Overweight (> 25.0)	34 (32)	26 (27)	60 (29)
	Obese (> 30.0)	28 (26)	22 (22)	50 (24)
Oral supplement intake	Yes/no	70 (65)	58 (59)	128 (62)
Oral supplement type^a^	Vitamins	28 (40)	17 (27)	45 (23)
	Minerals	21 (30)	15 (26)	36 (28)
	Pro/prebiotics	11 (16)	28 (48)	39 (30)
	Other	26 (37)	22 (38)	48 (38)
Current specific dietary regimen^a^	Gluten free	7 (7)	26 (27)	33 (16)
	Vegetarian	11 (10)	13 (13)	24 (12)
	Low FODMAP	0 (0)	14 (14)	14 (7)
	Vegan	10 (9)	12 (12)	22 (11)
	Mediterranean diet	15 (14)	4 (4)	19 (9)
	Low fat	5 (5)	5 (5)	10 (5)
	Low carb/keto	8 (7)	3 (3)	11 (5)
	EEN	9 (8)	0 (0)	9 (4)
	CDED	7 (7)	2 (2)	9 (4)
	Other diet	9 (8)	18 (18)	27 (13)
	No specific diet	38 (36)	19 (19)	57 (28)

*Note:* “Other” medications include omeprazole, sulfasalazine, amitriptyline, nortriptyline, antihistamines, and codeine. “Other” oral supplements include nutrient rescue, Athena protein + iron, beef bone broth, fish oil/omega‐3, Matakana green, flaxseed, Coenzyme Q10, olive oil, dried beef organs, creatine, Methylsulfonylmethane, digestive enzymes, broccoli sprout tabs, lysine, iodine, or Ashwagandha tabs. The “other diet” includes pescetarian, carnivore, lactose‐free, low‐residue, no sugar, IBD‐anti‐Inflammatory Diet (IBD‐AID), gut and psychology syndrome diet (GAPS), and dairy‐free diet. The variables are numbers (%) or median (IQR).

Abbreviations: BMI, body mass index; CD, Crohn's disease; CDED, Crohn's disease exclusion diet; EEN, exclusive enteral nutrition; FODMAP, fermentable oligosaccharides, disaccharides, monosaccharides, and polyols; IBDU, inflammatory bowel disease unclassified; IQR, interquartile range; *n*, number; UC, ulcerative colitis.

^a^ Participants could report more than one.

Overall, 128 (62%) participants with IBD reported regular intake of one or more oral supplements. Participants with CD reported greater use of oral multivitamins/mineral supplements, including vitamins, minerals, and other supplements, and less pre/probiotics compared with those with UC/IBDU χ ^2^ 15.3, *p* = 0.004, 95% CI (0.05, 0.13).

About two‐thirds of participants (*n* = 69; 64%) with CD and 79 (81%) with UC/IBDU followed one or more specific diets (Table 2). Twenty‐seven (13%) of the respondents adhered to “other diet.”

### 3.3 The Self‐Reported HRQoL of the Respondents

Overall, 142 (69%) of the respondents self‐reported impaired HRQoL. This was similar between those with CD (*n* = 74; 36%) and those with UC/IBDU (*n* = 68; 33%) (*p* > 0.05). Participants with active CD (mild/moderate) had a higher median total SHS score than those in remission (160; IQR: 117–236 vs. 73; IQR: 23–164, *p* = 0.001) (Table 3). Among the group with UC/IBDU, the median total SHS score was also higher in those with active disease than in individuals in remission (257; IQR: 178–301 vs. 115; IQR: 91–179; *p* = 0.001).

**TABLE 3:** Health‐related quality of life of 205 participants with IBD segregated by disease activity index.

		SCDAI		*p*	SCCAI		*p*
		Remission (*n* = 47)	Active CD (mild/moderate) (*n* = 60)		Inactive (*n* = 52)	Active (*n* = 46)	
HRQoL domains	System	10 (5–20)	31 (25–51)	< 0.001	10 (6–38)	69 (46–76)	< 0.001
	Function	15 (5–33)	40 (26–69)	< 0.001	10 (4–14)	71 (50–81)	< 0.001
	Worry	20 (5–60)	50 (26–64)	0.017	22 (10–42)	71 (50–89)	< 0.001
	Well‐being	23 (5–50)	49 (27–62)	0.050	70 (32–89)	54 (28–71)	0.669
	Total SHS score	73 (23–164)	160 (117–236)	< 0.001	115 (91–179)	257 (178–301)	< 0.001

*Note:* Scores expressed as medians (with interquartile ranges).

Abbreviations: CD, Crohn's disease; CI, confidence interval; HRQoL, health‐related quality of life; *n*, number; SCCAI, Short Clinical Colitis Activity Index; SCDAI, Short Crohn's Disease Activity Index; SHS, Short Health Scale; UC/IBDU, ulcerative colitis/inflammatory bowel disease unclassified.

### 3.4 Dietary Patterns of the Study Cohorts

The dietary patterns of this group of 205 adults with IBD were assessed using PCA. The sampling adequacy was acceptable (KMO = 0.60) and Barlett's test of sphericity demonstrated that correlations between items were large enough for PCA (*X* ^2^(df, 630) = 2138.2, *p* < 0.001). Sixteen food items were excluded from the analysis because they were consumed by < 10% of participants.

The PCA revealed six components that accounted for 51% of the variance. Component one explained the highest percentage of the variance (14.7%) with an Eigenvalue of 5.2, while the variance explained by component six was the lowest (4.9%) with an Eigenvalue of 1.7 (Table 4). Patterns were described based on the food groups that loaded most positively (factor loading > 0.30) under each respective component.

**TABLE 4:** Factor loadings of the six PCA orthogonal (varimax) rotation‐derived dietary patterns for 205 adults with IBD.

Component/pattern	Food groups	Eigenvalue	% variance explained	Factor loading
Western	Processed meat	5.2	14.7	0.7
	Sauces and condiments			0.6
	Sugary/diet soft/fizzy drinks			0.6
	Refined grains			0.6
	Snacks			0.5
	Refined vegetable oils			0.5
	Cereals			0.3
Vegetarian	Other vegetables	4.1	11.9	0.7
	Salad vegetables			0.7
	Drupes, pomes and berries			0.6
	Other fruits			0.5
	Alliums			0.5
	Green leafy vegetables			0.5
	Eggs, nuts, and seeds			0.4
	Whole grains			0.3
	Legumes			0.3
	Spices			0.3
Pescatarian	Soy‐based foods	2.5	6.9	0.9
	White fish/shellfish			0.8
	Green leafy vegetables			0.7
	Tea and coffee			0.5
	Sauces and condiments			0.3
	Sugary/diet soft/fizzy drinks			0.3
Semi‐pescatarian	Processed fish	2.3	6.8	0.7
	Whole grains			0.6
	Other alcoholic drinks			0.6
	Red wine			0.5
	Soup			0.5
	Processed meat			0.3
	Sauces and condiments			0.3
	Salad dressing			0.3
	Confectionary			0.3
Semi‐vegetarian	Root vegetables	1.8	5.7	0.9
	Legumes			0.9
	Banana			0.4
	Other vegetables			0.4
	Drupes, pomes and berries			0.3
	Eggs, nuts, and seeds			0.3
Low carbohydrate	Poultry	1.7	4.9	0.8
	Red meat			0.7
	Alliums			0.4
	Dairy products			0.4
	Soup			0.3

*Note:* Only food groups with factor loadings (≥ 0.3) are displayed.

### 3.5 Associations Between Dietary Patterns and Self‐Reported Disease Activity in Respondents With Crohn's Disease

After adjusting for medication use, comorbidities, and BMI, the Western dietary pattern was associated with increased odds of having moderate disease activity (AOR = 4.55, 95% CI (1.27, 7.26), *p* = 0.02) compared to other dietary patterns (Table 5). Participants who adopted a vegetarian or semi‐vegetarian dietary pattern had lower odds of having moderately active disease (AOR = 0.32, 95% CI (0.11, 0.98), *p* = 0.04) or (AOR = 0.33, 95% CI (0.12, 1.00), *p* = 0.04), respectively, in contrast with dietary patterns. The goodness‐of‐fit test showed a good model fit for this data (*χ* ^2^(df, 106) = 119.1, *p* = 0.183).

**TABLE 5:** Regression coefficients showing the relationship between dietary patterns and self‐reported Crohn's disease activity.

CDAI		*B*	AORs	*p*	95% CI	
					Lower bound	Upper bound
Mild	Intercept	−5.91		0.09		
	Western	0.96	2.61	0.06	0.98	6.92
	Vegetarian	−0.19	0.83	0.63	0.39	1.76
	Semi‐vegetarian	−0.01	0.99	0.98	0.52	1.87
	Pescatarian	0.65	1.92	0.42	0.39	5.37
	Semi‐pescatarian	0.61	1.84	0.35	0.51	6.57
	Low carbohydrate	0.19	1.21	0.47	0.72	2.01
Moderate	Intercept	−1.96		0.63		
	Western	1.514	4.55	0.02	1.27	7.26
	Vegetarian	−1.11	0.32	0.04	0.11	0.98
	Semi‐vegetarian	−1.12	0.33	0.04	0.12	1.00
	Pescatarian	0.87	2.37	0.34	0.39	6.21
	Semi‐pescatarian	0.83	2.29	0.27	0.52	5.18
	Low carbohydrate	−0.98	0.37	0.97	0.12	1.19

*Note:* The reference category was remission.

Abbreviations: AORs, adjusted odds ratios; *B*, coefficients; CDAI, Crohn's Disease Activity Index; CI, confidence Interval.

### 3.6 Associations Between Dietary Patterns and Self‐Reported Disease Activity in Respondents With Ulcerative Colitis/IBD‐Unclassified

After controlling for age, gender, medication use, and comorbidities, adherence to the Western dietary pattern was associated with increased odds of having active UC/IBDU (AOR = 3.50, 95% CI (1.07, 5.40), *p* = 0.04). Adherence to the semi‐pescatarian dietary pattern was also associated with higher disease activity (AOR = 3.48, 95% CI (1.05, 4.56), *p* = 0.04) (Table 6). There were no relationships between the pescatarian, vegetarian, or low‐carbohydrate dietary patterns and UC/IBDU activity (*p* > 0.05 for all). The model had a good fit and adequately described the data (*χ* ^2^(df, 8) = 2.28, *p* = 0.97).

**TABLE 6:** Regression coefficients for PCA‐derived dietary patterns and their association with self‐reported ulcerative colitis/inflammatory bowel disease unclassified activity.

	*B*	AOR	*p*	95% CI	
				Lower	Upper
Western	1.25	3.50	0.04	1.07	5.40
Vegetarian	−0.26	0.78	0.43	0.41	1.46
Pescatarian	0.84	2.32	0.43	0.28	8.75
Semi‐pescatarian	1.25	3.48	0.04	1.05	4.56
Semi‐vegetarian	0.047	1.05	0.93	0.39	2.83
Low carbohydrate	−0.55	0.57	0.25	0.22	1.49
Constant	−2.63	0.07	0.05		

*Note:* The reference category was inactive disease.

Abbreviations: AOR, adjusted odds ratio; *B*, coefficients; CI, confidence interval.

### 3.7 Relationship Between Dietary Patterns and Self‐Reported HRQoL of 205 Adults With IBD

The correlation between dietary patterns and the overall HRQoL (total SHS score) in participants with CD and UC/IBDU was assessed (Table 7). The model (*R* ^2^ = 0.51) explained 51% of the variance in HRQoL (*F* (10, 101) = 3.321, *p* < 0.001), indicating that dietary patterns were predictors of HRQoL. After controlling for IBD type and BMI, the Western dietary pattern (*B* = 32.8, *t* = 2.91, *p* ≤ 0.005, CI (2.9, 3.6)) or semi‐pescatarian pattern (*B* = 43.7, *t* = 3.23, *p* ≤ 0.002, CI (2.9, 3.6)) was associated with worse overall HRQoL. In contrast, there were no relationships between the vegetarian or low‐carb patterns and HRQoL (*p* > 0.05).

**TABLE 7:** Linear regression coefficients for the relationship between dietary patterns and HRQoL of adults with IBD, with controlling variables (number of observations = 205).

Model		*B*	SE	*β*	*t*	*p*	95% CI	
							Lower	Upper
1	(Constant)	123.37	45.35		2.72	0.008		
	Western	32.82	11.29	0.318	2.91	0.005	92.67	154.07
	Vegetarian	−15.13	8.83	−0.152	−1.71	0.090	25.17	40.46
	Pescatarian	29.67	19.75	0.144	1.50	0.136	−21.11	−9.15
	Semi‐pescatarian	43.67	13.51	0.332	3.23	0.002	16.29	43.03
	Semi‐vegetarian	−6.35	8.62	−0.067	−0.74	0.463	34.52	52.81
	Low carbohydrate	−3.55	7.81	−0.040	−0.46	0.650	−12.18	−0.51
	IBD type	56.10	17.32	0.300	3.24	0.002	−8.82	1.73
	BMI	−0.11	1.45	−0.005	−0.11	0.953	−35.53	−10.33

Abbreviations: *β* = standardized coefficients; *B* = unstandardised coefficients; BMI, body mass index; CI = confidence interval; SE = standard error; *t* = *t*‐statistics.

### 3.8 Foods or Drinks That Trigger Gut Symptoms

One hundred and ten participants (63 CD and 47 UC/IBDU) reported foods or drinks that trigger and/or aggravate their gut symptoms. Seventy‐six individual foods and 54 specific drinks were mentioned (Table S3). The most commonly reported food types to trigger gut symptoms were green leafy vegetables, allium species, and dairy products (Supporting Information Appendix A). Alcoholic beverages were the most commonly reported drink noted to trigger symptoms (Supporting Information Appendix B).

### 3.9 Foods or Drinks That Improve Gut Symptoms

Seventy‐five respondents (38 CD and 37 UC/IBDU) reported 31 specific foods and 22 drinks that help or improve their symptoms (Table S4). Mashed potato was most frequently mentioned, followed by rice and other foods (Supporting Information Appendix C). The major vegetables reported to ameliorate symptoms were stem vegetables, pumpkins, lettuce, tomatoes, carrots, capsicum, and spinach. Reportedly helpful fruits were kiwifruit, avocado, blueberries, grapes, cooked apples/apple puree, and stewed fruits. Among dairy products, yoghurt, kefir, and cheese were reported as being helpful. Herbal/organic teas and water were reported most commonly as beneficial drinks (Supporting Information Appendix D).

## 4 Discussion

This cross‐sectional study aimed to assess the current dietary patterns and food choices in a group of adults with IBD in NZ. The results show that the dietary patterns of this group of patients fitted into six categories. The predominant dietary patterns were Western, vegetarian, and pescatarian. Adherence to a Western or semi‐pescatarian pattern was associated with increased odds of self‐reported active disease (both CD and UC/IBDU) and predicted worse HRQoL, whereas vegetarian dietary patterns were protective. The patients were generally aware of specific foods or drinks that could produce or improve their gut symptoms.

The present study revealed diverse dietary patterns of adult New Zealanders with IBD. The patterns were, however, not mutually exclusive (i.e., some food groups loaded in more than one pattern). The food consumption patterns identified in the current study deviated in part from those of healthy adult New Zealanders, as reported in the NZ Health Survey [43]. The NZ Health Survey revealed two predominant eating patterns among healthy adults: the plant‐based and Western, which align with the current findings [43]. However, the patterns differed in regard to pescatarian and low‐carb patterns, which may reflect adherence to specific diets during remission or flare‐ups, and food avoidance.

A similar finding was reported in a study in the US, whereby the dietary patterns of patients with IBD differed partly from those of the healthy population [44]. In that study, the sugary consumption pattern (a typical characteristic of Western dietary patterns in the current study) was similar across groups, with a variation in the pescatarian and plant‐based patterns. It was evident that the patients with IBD ate healthier foods than the general population. This variance from the general population's food consumption pattern may suggest a desire to adopt a healthy dietary pattern to improve symptoms and overall well‐being.

Other dietary pattern studies among people with IBD have reported comparable outcomes, both within and outside NZ. One study in the Auckland region of NZ identified three dietary patterns among older adults with IBD, and although the study varied in scope and population from the current study, the Western dietary pattern was identical to that identified in the present study [45]. Similarly, Mediterranean and prudent patterns were comparable to the vegetarian and pescatarian patterns in the current study [45]. Studies conducted elsewhere (in the United States [46], Iran [47], and France [48]) are also consistent with the present findings, whereby reported dietary patterns (such as the Western and healthy dietary patterns) were either typical of or resembled the food consumption patterns identified in the current study. Overall, these findings reveal a commonality in the food consumption patterns of individuals with IBD across various countries or regions.

As seen in the current and previous works, the Western and plant‐based dietary patterns appear to be predominant among people with IBD. Analogous to the current findings, the Western dietary pattern in the IBD setting has been associated with disease exacerbation, while a plant‐based or vegetarian pattern has been linked to beneficial outcomes [49]. In the present study, people with inactive disease were more likely to follow a vegetarian dietary pattern. Existing evidence suggests that the Western dietary pattern affects IBD by inducing metabolic dysregulation and gut inflammation, altering gut microbial composition (dysbiosis), disrupting immune regulation, triggering oxidative stress, increasing intestinal permeability, activating psychological disorders, and dysregulating the gut‐brain axis [50, 51, 52, 53, 54, 55]. With regard to the plant‐based dietary pattern, mechanistic studies reveal that the pattern modulates the gut microbiota and promotes the production of short‐chain fatty acids [56, 57, 58, 59]. These, in effect, improve intestinal barrier function, reduce inflammation and the production of harmful metabolites as well as mitigate oxidative stress, and downregulate inflammatory signaling pathways [56, 57, 58, 59]. Despite the benefits of a plant‐based diet in ameliorating the course of IBD, the current study results show variable effects between those with CD and others with UC/IBDU. In the current study, the relationship between the vegetarian pattern and CD activity was strong (protective), but weak for UC/IBDU. Consistent with this finding, Nikniaz et al. [60] found no association between healthy plant‐based dietary index (hPDI) scores and UC activity. The food groups (e.g., nuts, legumes, fruits, vegetables, and whole grains) included in the hPDI group were similar to foods that loaded under the vegetarian patterns of the current study. Generally, the effects of a plant‐based diet in people with UC appear to depend on the quality of the plant‐based diet and timing [61].

Few studies have investigated the relationship between dietary patterns and HRQoL among people with IBD. The current study reports a strong influence of dietary patterns on HRQoL, specifically the Western and semi‐pescatarian dietary patterns being negatively associated with HRQoL. A previous systematic review linked healthy/Mediterranean dietary patterns with improved HRQoL, and unhealthy/Western patterns with higher (worse) HRQoL scores [62]. While most of the reviewed studies were conducted in the context of chronic diseases other than IBD, the dietary mechanisms in the pathophysiology of most chronic diseases, which later impact HRQoL, appear to be similar [63]. The relationship between the Western dietary pattern and HRQoL in IBD could relate to its role in driving gut inflammation and symptom exacerbation, leading to a decline in HRQoL [64].

Studies conducted in countries outside NZ have reported findings consistent with the current work on food choices related to symptom‐trigger or relief in individuals with IBD. Previous studies from Italy, Australia, and Canada revealed that people with IBD considered foods/drinks that trigger symptoms to include spicy foods, dairy products, carbonated drinks, legumes, fried foods, leafy vegetables, processed meat, and alcohol [65, 66, 67, 68]. The foods (fruits, vegetables, lactose‐free, gluten‐free, chicken, and fish) reported to alleviate symptoms were also similar between those studies and the current research.

Few prior studies have documented food choices in individuals with IBD in NZ. Congruent with the current results, a recent study by Yap et al. [69] reported food avoidance of dairy, vegetables, red meat, and gluten foods in 69% of participants with IBD. In that group, the food avoidance patterns correlated with a low intake of dietary calcium, selenium, magnesium, zinc, and iron. This finding has been confirmed by work carried out in South Korea, whereby patients avoiding milk/dairy products, legumes, meat, spicy or fried foods were deficient in calcium, vitamin A, and zinc intake [70]. The current study did not include any assessment of micronutrient intake: additional work would be required to elucidate this further. However, Triggs et al. [71] found contrasting results to the current work, whereby milk and leafy vegetables were reported to have beneficial effects for a group of people with CD. This could be due to differences in the study population and disease activity status. Consumption of cruciferous vegetables can be both protective and detrimental in CD/UC, depending on the current disease activity. During a flare‐up, the high insoluble fiber content in leafy vegetables could worsen symptoms as it may cause diarrhea, bloating, gas, and cramping [72, 73, 74, 75, 76]. This could explain why insoluble fiber‐rich foods (mainly from green leafy vegetables, raw fruits, and other cereals and nuts) were commonly reported as major triggers of gut symptoms in the current study. A soluble fiber‐rich diet may be advised for patients in active flares [77]. However, dietary fibers, including leafy vegetables, are well tolerated in patients with inactive IBD as they improve gut microbial diversity, bowel integrity, neutralize oxidative stress, and reduce inflammation [78, 79, 80].

The current study has some limitations. Data were collected using a dietary survey tool that may be prone to memory bias and inaccurate estimates of portion sizes. The recall bias was managed by indicating a specific time frame (e.g., “past three months”), rather than the standard recall period, “past months,” as it appears in the original REACH‐FFQ tool. There were more female participants than males, which may not accurately reflect the true gender distribution of the NZ population with IBD and likely demonstrates a degree of selection bias. Despite achieving adequate statistical power, the study sample size was relatively small.

This study had several strengths: First, it was a nationwide survey. This wider representation improved data reliability and generalizability of the study findings. Second, it collected detailed dietary data using a comprehensive and validated tool. This enabled the collection of reliable data on multiple variables to assess important exposure‐outcome relationships that could be of interest in future longitudinal studies. Third, it had approximately equal representation of IBD subtypes (CD or UC/IBDU), which enabled appropriate statistical comparisons between the IBD subtypes. This further contributed to the rigor and generalizability of results.

## 5 Conclusions

This study reports the current dietary patterns and food choices of adults with IBD in NZ and establishes relationships between dietary intake and disease activity and well‐being. The study findings may benefit clinicians or dietitians seeking to incorporate nutrition education and a personalized dietary plan with a psychosocial component in routine IBD care or management. This would allow for an intended goal of promoting balanced nutrition, addressing nutrient deficiencies, improving the outcomes and HRQoL of people with IBD.

In addition to benefiting clinicians, the present results may also be of value to patients seeking to improve their IBD symptoms through diet modification. The novel findings on the beneficial dietary patterns or food choices may help patients develop a personalized nutrition plan to improve their well‐being. However, where feasible, patients adapting to these should seek regular monitoring by a trained dietitian or healthcare provider to identify and minimize nutrient deficiencies associated with over‐reliance on a specific food pattern.

Further work is therefore warranted to evaluate an appropriate dietetic education approach, which could improve intake of beneficial foods and/or adherence to healthy consumption patterns in IBD.

## Funding

A.S.D.'s research activities are supported by Cure Kids.

## Ethics Statement

This study received ethical approval from the University of Otago Human Ethics Committee (Health) (H25/0484).

## Conflicts of Interest

The authors declare no conflicts of interest.

## Supporting information


**Table S1:** Food groups included in the final analysis.
**Table S2:** The food items excluded from the final analysis.
**Table S3:** Symptom trigger foods or drinks.
**Table S4:** Foods/drinks that improve IBD symptoms.

**Supporting Information: Appendix A.** The figure showing the frequency of occurrence and percentage of Top 10 foods reported by the participants to trigger gut symptoms.
**Supporting Information: Appendix B** The figure illusttrating the frequency of occurrence and percentage of five major drinks reported by the participants to aggravate gut symptoms.
**Supporting Information: Appendix C** The Figure representing the frequency of occurrence of 10 key foods reported to improve gut symptoms.
**Supporting Information: Appendix D** The Figure depicting the frequency of occurrence of five major drinks reported to improve gut symptoms.

## Data Availability

The data that support the findings of this study are available on request from the corresponding author. The data are not publicly available due to privacy or ethical restrictions.
