# Supplementary material for: Dietary Patterns and Food Choices of Adults With Inflammatory Bowel Disease in New Zealand
Source: JGH Open. 2026 Aug 9;10(8):e70457. doi: 10.1002/jgh3.70457 (PMC13454213; doi:10.1002/jgh3.70457)
Supplement: Supplementary file 1 [file JGH3-10-e70457-s001.docx]

**Appendices**

Supplementary Appendix A. The Figure showing the frequency of occurrence and percentage of top ten foods reported by the participants to trigger gut symptoms

|  | Frequency of reporting |
| --- | --- |
| Fried foods | 5 |
| Nuts | 6 |
| Corn | 10 |
| Red meat | 12 |
| Fruits | 14 |
| Gluten products | 15 |
| Spicy foods | 19 |
| Dairy products | 21 |
| Alliums | 23 |
| Green leafy vegetables | 24 |

Supplementary Appendix B. The Figure illusttrating the frequency of occurrence and percentage of five major drinks reported by the participants to aggravate gut symptoms

Frequency of reporting

|  | Occurrence |
| --- | --- |
| Fruit juice | 8 |
| Coffee | 20 |
| Fizzy drinks | 29 |
| Milk | 38 |
| Alcohol | 47 |

Supplementary Appendix C. The Figure representing the frequency of occurrence of 10 key foods reported to improve gut symptoms

|  | Occurrence |
| --- | --- |
| Mashed potato | 26 |
| Rice | 23 |
| Vegetables | 20 |
| Some fruits | 13 |
| Chicken | 10 |
| Eggs | 10 |
| Banana | 10 |
| Some dairy products | 10 |
| Fish | 9 |
| Kumara | 6 |

Supplementary Appendix D. The Figure depicting the frequency of occurrence of five major drinks reported to improve gut symptoms

|  | Occurrence |
| --- | --- |
| Herbal/organic tea | 27 |
| Water | 26 |
| Lactose-free milk | 8 |
| Plant-based milk | 8 |
| Ensure drinks | 4 |
