# Supplementary material for: Dietary Patterns and Food Choices of Adults With Inflammatory Bowel Disease in New Zealand
Source: JGH Open. 2026 Aug 9;10(8):e70457. doi: 10.1002/jgh3.70457 (PMC13454213; doi:10.1002/jgh3.70457)
Supplement: Supplementary file 2 [file JGH3-10-e70457-s002.docx]

**Supplementary materials**

**Supplemental Table 1. Food groups included in the final analysis**

| **Food groups (n = 36)** | **Food items (n = 93 (109-16)** |
| --- | --- |
| Bananas | Banana |
| Leafy cruciferous vegetables | Green leafy vegetables, e.g. spinach, silver beet, Swiss chard, watercress, puha, whitloof, chicory, kale, chard, collards, Chinese kale, bok choy, taro leaves (palusami), Broccoli, cauliflower, Brussel sprouts, cabbage (all varieties). |
| Alliums | Onions, leeks, garlic |
| Red meat | Beef, lamb, hogget, mutton, pork, veal, e.g., roast, steak, fried, chops, schnitzel, silverside (rump roast), casserole, stew, stir fry, curry, BBQ, hamburger meat, mince dishes, frozen dinners. |
| Poultry | Chicken, turkey or duck, e.g., roast, steak, fried, steamed, BBQ, casserole, stew, stir fry, curry, mince dishes, frozen dinners |
| Processed meat | Ham, bacon, luncheon sausage, salami, pastrami, other processed meat', 'sausages, frankfurters, Cheerios (cocktail sausage), hot dogs |
| White fish and shellfish | Shellfish, e.g., cockles, kina, oysters, paua, scallops, shrimp/prawn, pipi, roe, 'tuna (canned), hoki, gurnard, hake, kingfish, cod, tarakihi, groper, flounder |
| Soy-based foods | Tofu, soybeans, tempeh |
| Refined grains | White bread and rolls, including sliced and specialty breads such as foccacia, panini, pita, naan, chapatti, ciabatta, Turkish, English muffin, crumpets, pizza bases, wraps, tortillas, burrito, roti, rewena bread, white pasta, noodles, e.g. spaghetti, canned spaghetti, vermicelli, egg noodles, rice noodles, instant noodles, white rice |
| Snacks | Crackers eg, crisp bread, water crackers, rice cakes, cream crackers, Cruskits, Meal Mates, Vita-wheat, muesli or cereal bar (all varieties) |
| Whole grains | Brown rice, couscous, polenta, congee, Bulgur wheat, quinoa eg, tabbouleh, whole-grain or multigrain bread and rolls, including sliced and specialty breads, whole-meal or wheat -meal bread and rolls, including sliced and specialty breads, whole-meal pasta, noodles |
| Tea and coffee | Coffee (all varieties), herbal tea, fruit tea, tea |
| Juice | Fruit and vegetable juices (all varieties) |
| Water | Water, including tap, bottled or sparkling water |
| Red wine | Red wine |
| Vegetable oils | Margarine, vegetable oils |
| Yeast spreads | Marmite, Vegemite |
| Salad dressings | Creamy dressings, e.g., mayonnaise, tartar, thousand island, ranch dressing, light dressings e.g., French and Italian dressing, balsamic vinegar. |
| Sauces and condiments | Pickles, chutney, mustard, tomato sauce, barbecue sauce, sweet chilli sauce, white sauce, cheese sauce, gravies |
| Spices | Spices e.g. turmeric, ginger, cinnamon |
| Soup | Soup, homemade or canned |
| Sugary/diet/fizzy drinks | Cordials (non-alcoholic fruit-flavored concentrates) energy drinks eg, Red Bull, V, hot chocolate, drinking chocolate, cocoa, Ovaltine, Nesquik, Milo, soft/fizzy drinks eg, Sprite®, Coke, Sprite Zero, Diet Coke, Coca-Cola Zero Sugar, |
| Other milk/non-dairy products | Soy milk, coconut milk, rice milk, almond milk, coconut cream |
| Dairy products | Cheese eg, Cheddar, Colby, Edam, Tasty, blue vein, camembert, parmesan, gouda, feta, mozzarella, brie, processed, cottage cheese, ricotta cheese, Cream, sour cream, cream cheese, cheese spreads, Cow’s milk, including milk as a drink, milk added to drinks (eg, milky coffees), milk added to cereal, Ice cream, smoothies, milk shakes (made from milk, yoghurt, ice cream), flavoured milk, Yoghurt, Butter, ghee |
| Other alcoholic drinks | Beer, lager, cider (hard) (all varieties), Spirits eg, gin, brandy, whiskey, vodka, white wine |
| Salad vegetables | Salad vegetables eg, lettuce, cucumber, celery, sprouts, Tomatoes (all varieties) |
| Legumes | Green beans, broad beans, runner beans, peas, green, Beans (canned or dried) eg, black beans, butter beans, haricot beans, kidney beans, cannellini beans, refried beans, baked beans, chilli beans, peas and lentils eg, chickpeas, hummus, falafels, split peas, cow peas, dahl |
| Root vegetables | Carrots, Kumara, taro, green banana, cassava, e.g., boiled, mashed, baked, roasted, other root vegetables, e.g., yams, parsnip, swedes, beetroot, turnips, potato, e.g., boiled, mashed, baked, jacket, instant, roasted |
| Other fruits | All other fruit eg, feijoa, persimmon, tamarillo, kiwifruit, grapes, mango, melon, watermelon, pawpaw, papaya, pineapple, rhubarb, Dried fruit eg, sultanas, raisins, currants, figs, apricots, prunes, dates, Citrus fruits eg, orange, tangelo, tangerine, mandarin, grapefruit, lemon, lime |
| Drupe, pome, and berry | Apples, pears, nashi pears, Avocado, olives, Strawberries, blackberries, cherries, blueberries, boysenberries, loganberries, cranberries, gooseberries, raspberries (fresh, frozen, canned) |
| Confectionary | Jam, marmalade, honey, syrups, sweet spreads or preserves, sugar (all varieties) added to food/drinks, sweets, lollies, biscuits (cookies), plain; cakes, slices (slabs), pastries |
| Chocolates and puddings | Chocolate (all other varieties), Biscuits (cookies), chocolate or cream filled, non-milk based puddings eg, pavlova, sweet pastries, fruit pies, trifle; pancakes, waffles, sweet buns, scones, sweet muffins, fruit bread, croissants, doughnuts, brioche |
| Processed fish | Fish fried in butter (from fish & chips shop), Albacore tuna, salmon, sardines, herring, kahawai, swordfish, carp, dogfish, gemfish, alfonsino, rudderfish, anchovies, mackerel, snapper, oreo, barracouta, trevally, dory, trout, eel |
| Other vegetables | All other vegetables e.g., corn, pumpkin, mushrooms, capsicum, peppers, courgette, zucchini, gherkins, marrow, squash, asparagus, radish, eggplant, artichoke', |
| Cereals | Bran based cereals, muesli, porridges – eg, rolled oats, oat bran, oatmeal, All-Bran, Sultana Bran, Other breakfast cereals eg, Special K, Light n Tasty, Weet-Bix, cornflakes, or rice bubbles |
| Egg, nuts, and seeds | Eggs – boiled, poached, raw, eggs - fried, scrambled, egg-based dishes including quiche, soufflés, frittatas, omelets, Nut butters or spreads eg, peanut butter, almond butter, pesto, nuts eg, peanuts, mixed nuts, macadamias, pecans, hazelnuts, brazil nuts, walnuts, cashews, pistachios, almonds, seeds eg, pumpkin seeds, sunflower seeds, pinenuts, sesame seeds, tahini |

**Supplemental Table 2. The food items excluded from the final analysis**

**Food items**

Stone fruits (e.g., apricots, nectarines, peaches, plums, lychees)

Liver

Kidney

Other offal (including pate)

Corned or canned beef or povi masima

Pork bones and/or lamb flaps

Crumbed fish (e.g., patties, cakes, fingers, nuggets)

Green mussels and squid

Vegetarian sausages/meat (e.g., vegetarian burger patty, textured vegetable protein)

Sweetened cereals (e.g., Nutrigrain, Fruit Loops, Honey Puffs, Frosties, Milo cereal)

Milk-based puddings (e.g., rice pudding, custard, semolina, instant puddings)

Low-calorie cordials

Cordials including syrups and powders (e.g., Raro)

Port, sherry, and liquors

Ready-to-drink alcoholic beverages (e.g., Pals, Long-white, or Gordon’s)

Coconut oil

**TOTAL = 16**

**Supplemental Table 3. Symptom trigger foods or drinks**

| Foods | | Drinks | |
| --- | --- | --- | --- |
| Group | **List** | **Group** | **List** |
| Green leafy vegetables | Cabbage, cauliflower, lettuce, broccoli, kale, silverbeet, and celery | Fruit juice | Apple juice, orange juice, |
| Alliums | Onions, garlic, shalots, leeks | Coffee |  |
| Dairy products | Cream, some cheese, and butter | Fizzy drinks | Energy drinks, sodas, diet drinks, |
| Spicy foods |  | Milk | Full cream milk, milkshakes, |
| Gluten products | Wheat, | Alcohol | Wine, beers, |
| Fruits | Cirus fruits, raw apples, oranges, raw pears, raspberries, watermelon, mango, | Others | Other sugary drinks, artificial sweeteners, |
| Red meat |  |  |  |
| Corns | Sweet corns, popcorns, |  |  |
| Nuts |  |  |  |
| Fried foods |  |  |  |
| Others | Grains, tomatoes, beans, hot chilli, pie, chips, burgers, Chocolate, pizza, pastas, linseed/flaxseed, artificial sweetener, Muesli, sausages, high-fat foods, honey, |  |  |

A word frequency of ≥5 for either improvement or worsening of symptoms was required for the food item to be included in the table.

**Supplemental Table 4. Foods/drinks that improve IBD symptoms**

| Foods | | Drinks | |
| --- | --- | --- | --- |
| Group | **List** | **Group** | **List** |
| Potato | Mashed potato | Herbal/organic tea | Green tea, lemonade, Peppermint tea, chamomile, Ginger tea, Nettle leaf tea, lemon tea, turmeric tea, mint tea, organic matcha, and Aloe vera juice |
| Rice | Mainly white rice | Water |  |
| Vegetables | Rooty vegetables, stem vegetables, pumpkins, Lettuce, Tomato, Carrot, Capsicum, carrot, and spinach. | Lactose-free milk |  |
| Some fruits | Kiwifruits, avocado, blueberries, grapes, cooked apples/apple puree, and stewed fruits, | Plant-based milk | Almond milk, Soy milk, oat milk, rice milk, coconut milk, |
| Chicken |  | Ensure drinks |  |
| Eggs |  | Other | Smoothies, bone broth, prebiotic drinks, powerage, |
| Banana |  |  |  |
| Some dairy products | Yoghurt, cheese, and kefir |  |  |
| Fish | Salmon, tuna, |  |  |
| Kumara | Mashed form |  |  |
| Others | Broth, soup, gluten-free bread, lentils, oats, gingernuts, Miso, white foods, custards, roux, |  |  |

Word sizes denote the frequency of occurrence in the text
